# Investigation into the Presence of and Serological Response to XMRV in CFS Patients

**DOI:** 10.1371/journal.pone.0017592

**Published:** 2011-03-09

**Authors:** Otto Erlwein, Mark J. Robinson, Steve Kaye, Gillian Wills, Shozo Izui, Simon Wessely, Jonathan Weber, Anthony Cleare, David Collier, Myra O. McClure

**Affiliations:** 1 Jefferiss Research Trust Laboratories, Section of Infectious Diseases, Wright-Fleming Institute, Faculty of Medicine, Imperial College London, London, United Kingdom; 2 Department of Pathology and Immunology, Faculty of Medicine, University of Geneva, Geneva, Switzerland; 3 Department of Psychological Medicine, Institute of Psychiatry, King's College London, Camberwell, London, United Kingdom; 4 Social Genetic and Developmental Psychiatry Centre, Institute of Psychiatry, King's College London, London, United Kingdom; National Institute of Allergy and Infectious Diseases, United States of America

## Abstract

The novel human gammaretrovirus xenotropic murine leukemia virus-related virus (XMRV), originally described in prostate cancer, has also been implicated in chronic fatigue syndrome (CFS). When later reports failed to confirm the link to CFS, they were often criticised for not using the conditions described in the original study. Here, we revisit our patient cohort to investigate the XMRV status in those patients by means of the original PCR protocol which linked the virus to CFS. In addition, sera from our CFS patients were assayed for the presence of xenotropic virus envelope protein, as well as a serological response to it. The results further strengthen our contention that there is no evidence for an association of XMRV with CFS, at least in the UK.

## Introduction

Hitherto, gammaretroviruses have never been found in human populations In mice, for example, the murine leukemia viruses (MLVs) which belong to this genus of retroviruses can be both exogenous and endogenous. Classically, endogenous MLVs are categorised on the basis of their envelope gene sequence and their cognate receptor polymorphism into ecotropic (replicating only in murine cells), xenotropic (replicating in multiple mammalian cells, including human but not murine cells), polytropic and modified polytropic (able to grow in murine and nonmurine cells) [1].

The discovery by microarray technology of a retrovirus in human prostates that was most closely related in its sequence to the xenotropic MLVs explains its designation as xenotropic murine leukemia virus-related virus (XMRV) [2]. Several reports have since confirmed the XMRV association with prostate cancer in the US [3]–[5], but not in Europe with the exception of one study in Germany study in which it was found in one patient and one control [6]–[8]. One Mexican study described the identification of XMRV sequences in one healthy control, but not in prostate tumor [9].

Interest in XMRV heightened when a study from the Whittemore Peterson Institute, published in *Science* by Lombardi et al [10] identified XMRV in 67% of patients suffering from chronic fatigue syndrome (CFS) and in 3.7% of healthy controls. One [11] of the many independent studies that failed to find XMRV in a variety of CFS patient cohorts [12]–[17] amplified instead genetic sequences closely related to the polytropic MLVs. We [12] among others failed to confirm the presence of XMRV in CFS patients tested not only in Europe [13], [14] and China [15] but also in the US [16], [17] where the original study was carried out. It has been suggested that these negative results could have arisen because of a failure to duplicate the experimental conditions described in the original publication by Lombardi et al [10]. To remove any element of doubt, we have repeated our genetic analysis using the same oligonucleotide primer sets described in [10] and, in addition, we have assayed sera taken from 130 of our CFS patients and 30 normal healthy subjects (NHS) for antibodies to the New Zealand Black (NZB) xenotropic virus and its envelope protein, gp70, a virus that shares more than 94% overall homology with XMRV.

## Materials and Methods

### Patient samples

All patients gave written informed consent for the use of their DNA to test aetiological theories of CFS, and the study was approved by the South London and Maudsley NHS Trust Ethics Committee. The patient samples have been described in detail previously [12]. Briefly, patients were recruited into our study from consecutive referrals to the CFS clinic at King's College Hospital, London and constitute a well-characterised and representative sample of routine clinic attenders. All patients had undergone medical screening to exclude detectable organic illness, including a minimum of physical examination, urinalysis, full blood count, urea and electrolytes, thyroid function tests, liver function tests, 9 a.m. cortisol and ESR. Patients were interviewed using a semi-structured interview [18]; all included patients met the CDC international consensus criteria for CFS [19]. Patients with the Fukuda-specified exclusionary psychiatric disorders, or somatisation disorder (as per DSM-IV), were not included. The use of standardised scales revealed high levels of fatigue and disability (see [12] and supplementary correspondence for further details). Overall we are confident that the patients in this study are typical of those seen in secondary care and/or CFS clinics around the world [20].

### PCR amplification of XMRV sequences

DNA extraction has been described previously [12]. Briefly, DNA was extracted from whole blood collected in EDTA. As a control for DNA integrity, a 124 nucleotide (nt) section of the human beta-globin gene was amplified. Amplification of XMRV *gag* sequences was carried out using primers, 419F and 1154R, and of *env* sequences using primers, 5922F and 6273R [10].

Reactions were carried out in a volume of 25 µl which contained 0.5 units TaqGold (Applied BioSystems, Warrington, UK), 1× TaqGold reaction buffer (Applied BioSystems), 1.5 mM Mg++, 200 mM each dNTP, 2.5 pmol each primer to which 5 µl DNA extract or control (see below) was added. The PCR conditions were as follows; one cycle at 94°C for 8 minutes, 45 cycles at 94°C for 30 seconds, 55°C for 30 seconds 72°C for 1 minute and a final annealing cycle of 72°C for 7 minutes. A 5 µl aliquot of the PCR product was applied to a 1% agarose gel, stained with Ethidium Bromide and subjected to electrophoresis. Each PCR included six no-template (water) controls and a positive control, plasmid pXMRV, containing the full-length XMRV isolate, vp62, for which we are grateful to R. Silverman.

### Serology

Sera from 130 CFS patients and 30 normal healthy subjects (NHS) as controls were assayed blind and in duplicate for antibody responses to the xenotropic NZB retrovirus envelope protein, gp70 by two independent ELISAs, based on antibodies against the NZB virus and the ecotropic Rauscher MLV. For the assays, a CNBr-activated Sepharose 4B column coupled with gp70-enriched serum glycoproteins was used to affinity-purify either goat anti-NZB xenotropic virus antibodies, or goat anti-Rauscher MLV gp70 antibodies, as previously described [21].

### gp70 antigen capture ELISA

Microtiter plates were coated with 83A25 rat IgG2a anti-retroviral gp70 mAb (10 µg/ml), which recognizes xenotropic, polytropic, ecotropic and amphotropic murine leukemia viruses [22]. Serum samples were diluted 1∶100 in PBS containing 1% BSA and 0.05% Tween 20 and incubated overnight at 4°C. Virus was detected following a 5-hour incubation with affinity-purified goat anti-Rauscher MLV gp70 or affinity-purified goat anti-NZB xenotropic virus antibodies, labelled with alkaline phosphatase. Results are expressed as absorbance values at 405 nm (A_405_). In these assays, 2-fold serially diluted NZB virus, obtained from ViroMed Biosafety Laboratories, Camden, NJ, was used as a positive control.

### XMRV antibody response

Serum IgG anti-XMRV responses were determined by ELISA. Microtiter plates were coated with whole inactivated NZB retrovirus (10 µg/ml), and incubated with 1∶100 diluted serum samples overnight at 4°C. The assay was developed following a 5 hour incubation with alkaline phosphatase-labelled goat anti-human IgG antibody (Cappel Laboratories, Durham, NC). The A_405_ values are shown. As there is no defined XMRV-positive human antiserum available, we used 603, a murine anti-Xeno gp70 monoclonal antibody [23] as a positive control.

### Statistical analysis

Data were analysed by unpaired Student's T test comparison of absorbance values between CFS patients and NHS controls. The ELISA cut-off was calculated as the mean + 3 standard deviations from the A_405_ value of the NHS sera.

## Results

**Figure 1:**
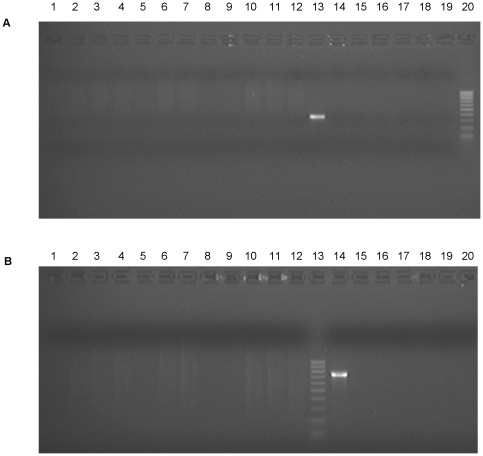
PCR amplification for XMRV in CFS patients. (**A**) Amplification products from nested PCR using XMRV *env* primers are shown. The *env* specific product which is 352 nt long was not amplified from CFS patients 37–48, as shown in lanes 1–12, but was amplified from the plasmid pXMRV, lane 13. The no-template controls are shown in lanes 14–19 and the DNA marker in lane 20. (**B**) The *gag* specific product, 736 nt in length, was not amplified from the same CFS patient DNA samples, lanes 1–12. The DNA marker is shown in lane 13, the plasmid pXMRV in lane 14 and the water controls in lanes 15–20.

We examined 48 of the previously described [12] 186 samples by PCR using primers derived from the *gag* and *env* regions of the XMRV genome previously published by Lombardi et al [10]. No amplicons were observed in either reaction (**Figures 1a and 1b**).

**Figure 2:**
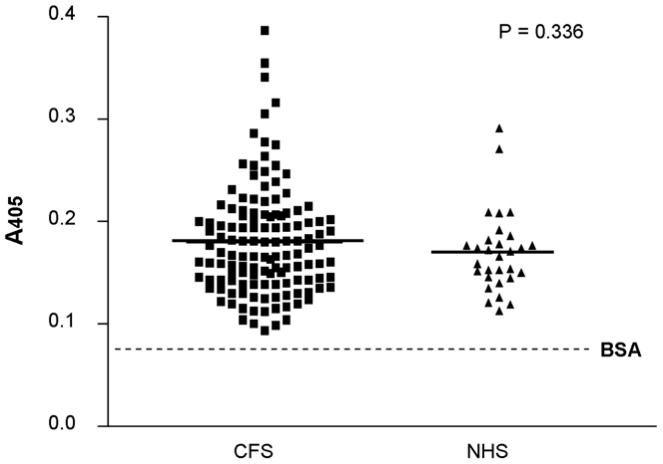
Antibody ELISA using sera from CFS patients and normal health subjects (NHS). The absorbance A_405_ values for 130 sera from CFS patients and NHS are shown. For the antibody ELISA whole NZB xenotropic virus was coated to the microtitre plate following incubation with serum samples overnight. Antibodies were detected by incubation with anti-human IgG antibodies labelled with alkaline phosphatase. The solid lines represent the mean. The BSA background of 0.071 is represented by the dotted line.

Sera from 130 CFS patients and 30 NHS controls were assayed by ELISA for antibodies to MLV Env protein and in an antigen capture ELISA for the presence of MLV Env itself. Results are shown in **Figures 2** **and** **3**.

**Figure 3:**
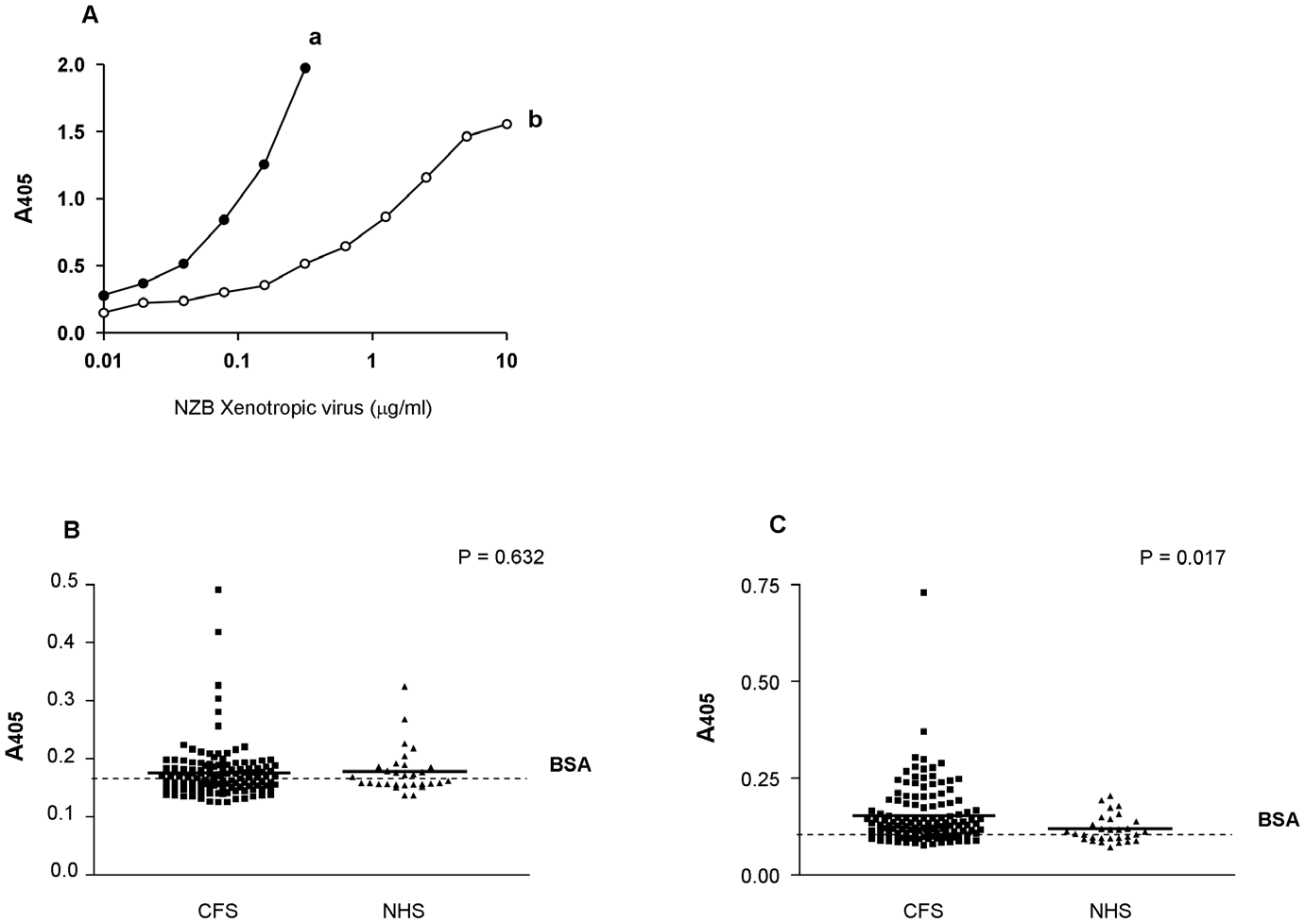
Antigen-capture ELISA using sera from CFS patients and NHS. (**A**) Goat anti-Rauscher MLV gp70 (lane a) and goat anti-NZB xenotropic virus (lane b) antibodies were tested for their reactivity against NZB xenotropic virus in 2-fold serial dilutions starting with 10 µg/ml. The A_405_ values are given. For the antigen capture ELISA, xenotropic virus envelope was captured onto the microtitre plate using 83A25 rat monoclonal antibody [22]. Detection was then carried out using goat anti-Rauscher antibodies or goat anti-NZB xenotropic MLV gp70 antibodies. (**B**) Results using goat anti-Rauscher MLV gp70 antibodies. The solid lines represent the mean and the dotted line indicates the BSA background of 0.161. (**C**) Results shown for goat anti-NZB xenotropic virus antibodies. The solid line represents the mean and dotted line indicates the BSA background of 0.095.

For the antibody ELISA, NZB virus was coated onto microtiter plates and patient sera incubated at a dilution of 1∶100. The reaction was detected by alkaline phosphatase-labelled polyclonal goat anti-human IgG antibody. The A_405_ value obtained with BSA as background was 0.071. The cut-off was at 0.289. No specific serological reaction could be detected (*p* = 0.336, **Figure 2**).

For the antigen capture ELISA, 83A25 rat IgG2a antibody raised against murine leukaemia virus envelope protein and able to recognise xenotropic, polytropic, ecotropic and amphotropic MLV [22], was coated onto microtiter plates and the serum added at a dilution of 1∶100. On Western blots this antibody recognised recombinant XMRV envelope protein, gp70, produced in 293 T cells (data not shown). To detect bound Env protein, affinity-purified polyclonal antibodies, goat anti-Rauscher MLV gp70 or goat anti-NZB xenotropic virus labelled with alkaline phosphatase were employed. These antibodies had demonstrated reactivity to NZB virus in experiments using 2-fold serial dilutions (**Figure 3a**). When goat anti-Rauscher MLV gp70 antibody was used as an XMRV detection reagent on sera from CFS patients and NHS (**Figure 3b**), the background A_405_ value obtained with BSA was 0.161. The cut-off value was 0.296. Four CFS samples and one NHS sample gave A_405_ values above the cut-off range, but the reactivity was not significant (*p* = 0.632).

When using goat anti-NZB xenotropic virus antibody, the A_405_ value of BSA was of 0.095. Twenty CFS sera were significantly above the cut-off value of 0.222, *p* value of 0.017 (**Figure 3c**). However, the reactivity was not demonstrated in both ELISAs (**Table 1**). For example, the CFS sera 122 and 186, which displayed the highest A_405_ values of 0.730 and 0.371, respectively, in the goat anti-NZB virus antibody ELISA, had A_405_ values of 0.159 and 0.138 in the goat anti-Rauscher gp70 MLV ELISA, a value below the BSA background of 0.161. Moreover, CFS sera 261 and 61, which showed the highest absorption of o.492 and 0.419 in the goat anti-Rauscher gp70 ELISA, gave values of 0.181 and 0.167 (both below the cut-off value of 0.222) in the anti-NZB virus antibody ELISA.

**Table 1:** Absorbance values of CFS sera in the two antigen-capture ELISAS used.

	ELISA A_405_	
CFS patient number	α-Rauscher	α-NZB
17	0.304	0.299
61	0.419	0.167
261	0.492	0.181
185	0.327	0.278
122	0.159	0.730
186	0.138	0.371

α-Rauscher, goat anti-Rauscher MLV antibody.

α-NZB, goat anti-NZB antibody.

The values are the mean of two independent experiments. α -Rauscher, goat anti-Rauscher gp70 MLV antibody; α-NZB, goat anti-NZB virus antibody.

Only sera 17 and 185 produced A_405_ values of 0.304 and 0.327, slightly above the cut-off of 0.296 in the goat anti-Rauscher MLV antibody ELISA. These sera also had a modest reaction of 0.299 and 0.278 in the goat anti-NZB virus antibody ELISA (cut-off being 0.222). However, as other samples showed similar absorbance values in this assay that did not significantly react in the goat anti-Rauscher gp70 MLV ELISA, the reactions are considered to be non-specific. Taken together, these experiments suggest that neither XMRV nor a specific serological response against this family of viruses was found in CFS patients.

## Discussion

The connection between XMRV and CFS remains highly controversial. The initial report by Lombardi et al [10] identified XMRV in 67% of CFS patients and 3.7% of health control subjects. Subsequent to this, using the same experimental protocol an independent study by Lo et al. [11] detected four classes of MLV-related *gag* sequences in 86.5% of CFS patients and blood donors. In this study [11] it was suggested that MLV-related sequences found in CFS cases vary to the extent that they may have remained undetected by our original PCR primers which targeted the leader *gag* region and the *pol* region of XMRV. We have now used the primers described in the original paper [10] which bind to the XMRV *gag* and *env* open reading frames. We have again failed to detect XMRV or MLV-related sequences in 48 of our CFS patients, demonstrating that our failure to find XMRV in CFS tissue is not a reflection of the primers used in the amplification process. Indeed, the original primer sets have been exploited in several studies which have failed to amplify XMRV sequences in CFS patient samples [14], [16], [17].

We have extended our study to investigate any possible serological response to XMRV in our CFS patients, since there appears to be a mis-match between PCR data and serology [10], [24]. In the absence of a specific XMRV serological assay, we made use of an ELISA in which Goat anti-Rauscher MLV gp70 and goat anti-NZB xenotropic virus antibodies were employed to detect gp70 Env protein in sera of CFS patients. In control experiments these antibodies clearly recognised recombinant XMRV gp70 Env expressed on dog thymus D17 cells (data not shown). Both assays highlighted samples with A_405_ values above the cut-off, however, for the goat anti-Rauscher MLV gp70 ELISA, this result was not significant. Using the goat anti-NZB xenotropic virus ELISA, twenty sera were above the cut-off, but most of these sera cluster outside the linear range with low absorbance values at the limit of detection of the assay as indicated by the flat curve b in **Figure 3a**. Considering further the fact that these sera did not behave consistently in both ELISAs, we believe that no specific serological reactions could be detected in the sera of the CFS patients.

Non-specific serological reactions have also been described by Switzer et al in an ELISA using recombinant XMRV Env protein [16]. In this case, one of 51 sera from CFS patients and one from 53 healthy controls were slightly above the cut-off, but both sera were negative by further testing by immunofluorescence assays and PCR. Groom et al [14] detected some neutralisation activity against MLV particles, pseudotyped with XMRV Env. This neutralisation activity was also present in healthy blood donors and showed no correlation with CFS [14], nor could XMRV specific sequences be detected by PCR in these samples.

It would not be inconceivable that our failure to replicate was due to patient differences. Clinical details of patients in the original study [10] remain sparse. By contrast, we provide evidence confirming that our patients not only had CFS, but had considerable disability, and were not contaminated by either exclusionary psychiatric or medical diagnoses. While it is true that there will always be some variation based on local referral practices, differences in health care provision and so on, and while it is also true that CFS remains a difficult and elusive construct, even when international consensus criteria are applied, we doubt that this could explain the full range of differences in prevalence of XMRV published to date.

Our serological data are consistent with the idea that XMRV may be present in the human population at some level [14], [16]. However, this study confirms our previous report that a connection of XMRV or MLV-related viruses with UK CFS patients could not be substantiated.
